# Microbiological PCR Characteristics of Odontogenic Sinusitis and Their Clinical Correlates: A Cross-Sectional Analysis

**DOI:** 10.3390/jcm15051814

**Published:** 2026-02-27

**Authors:** Marta Aleksandra Kwiatkowska, Alicja Trębińska-Stryjewska, Dariusz Jurkiewicz, Elżbieta Anna Trafny

**Affiliations:** 1 Department of Otolaryngology and Oncological Laryngology with Division of Cranio-Maxillo-Facial Surgery, Military Institute of Medicine—National Research Institute, 04-141 Warsaw, Poland; 2 Biomedical Engineering Centre, Institute of Optoelectronics, Military University of Technology, 00-908 Warsaw, Poland; alicja.trebinska@wat.edu.pl (A.T.-S.);

**Keywords:** odontogenic sinusitis, microbiology, polymerase chain reaction, endoscopic sinus surgery, periapical lesion

## Abstract

**Background**: Odontogenic sinusitis (ODS) represents a distinct form of maxillary sinus inflammation arising from dental pathology and is most commonly unilateral. Despite its polymicrobial nature and predominance of anaerobic organisms, molecular characterization of the bacterial profile and its relationship to clinical severity remains limited. This study aimed to evaluate associations between targeted quantitative PCR (qPCR) findings from paired maxillary sinus and periapical lesion samples and clinical, endoscopic, and radiological features of disease. Additionally, the influence of oroantral communication on microbial concordance between odontogenic and sinus sites was examined. **Methods**: Twenty-eight patients with confirmed ODS were included for analytical cross-sectional study and underwent combined otolaryngological and dental assessment. During endoscopic sinus surgery with extraction of the causative tooth, paired specimens were collected from sinus mucosa and periapical lesions under sterile conditions and preserved for molecular analysis. Targeted qPCR assays using 16S rRNA–based primers were performed to detect predefined odontogenic pathogens. Associations between bacterial detection patterns and clinical, endoscopic, and imaging variables were analyzed. **Results**: Detection of Streptococcus anginosus group organisms was significantly associated with complete maxillary sinus opacification. *Fusobacterium nucleatum* and *Porphyromonas endodontalis* demonstrated higher detection rates in patients with more advanced radiological disease, although statistical significance was not reached. Purulent nasal discharge correlated with detection of *Fusobacterium nucleatum*, *Porphyromonas endodontalis*, and streptococcal species. Cases with intraoperative oroantral communication exhibited greater concordance between sinus and dental microbial profiles. **Conclusions**: ODS is characterized by a polymicrobial environment dominated by anaerobic bacteria, with specific organisms associated with markers of disease severity such as purulent secretion and extensive sinus opacification. Targeted molecular profiling may improve recognition of odontogenic origin and support individualized therapeutic strategies, although larger studies integrating clinical outcomes are required to clarify prognostic implications.

## 1. Introduction

Chronic rhinosinusitis (CRS) is a complex inflammatory condition of the paranasal sinuses, often categorized into subtypes based on etiology and underlying pathophysiology [1]. Odontogenic sinusitis (ODS) is a subtype of CRS caused by dental infections, typically involving maxillary sinus (MS). This condition is increasingly recognized as a significant portion of maxillary sinusitis cases, with studies estimating its prevalence up to 40% in certain cohorts [2,3]. ODS is often associated with polymicrobial infections, with anaerobic bacteria such as *Fusobacterium nucleatum* and *Porphyromonas gingivalis* predominating [4,5,6]. These bacteria are also implicated in periodontal disease, reflecting the close anatomical and pathological link between dental structures and MS. This direct anatomical and pathological connection between the periapical lesions (PAL) of the posterior maxilla and the maxillary sinuses often complicates diagnosis and management, as the disease process involves both dental and otolaryngological domains.

The pathophysiology of ODS is thought to involve the disruption of the Schneiderian membrane by infectious or inflammatory stimuli originating from PALs, leading to sinus mucosal changes and secondary bacterial colonization [7,8]. Previous studies rarely have explored the microbiological profiles of ODS using advanced molecular methods like PCR and high-throughput sequencing. These methods highlight the predominance of oral anaerobes, including *Prevotella*, *Streptococcus constellatus*, and *Porphyromonas*, alongside facultative anaerobes like *Staphylococcus aureus* [9,10]. While some research has focused on identifying bacterial species in sinus aspirates or dental samples [5,11,12,13], correlations between bacterial presence and clinical variables—such as radiological severity, age, gender, BMI, or presence of oroantral communications—remain underexplored. 

The diagnosis of ODS relies heavily on a combination of clinical, endoscopic, and radiological findings. Advanced imaging modalities such as cone-beam computed tomography (CBCT) have proven to be particularly useful for identifying dental origins of infection and assessing the extent of sinus involvement [14,15]. Radiological scoring systems, including the Lund–Mackay and Zinreich scales, offer structured methods for evaluating disease severity and have been adapted for research purposes to correlate imaging findings with clinical outcomes [1,16,17]. 

Despite the growing understanding of ODS, uncertainty remains in delineating the relationship between bacterial presence and clinical or radiological severity. Furthermore, the impact of anatomical disruptions, such as oroantral communication (OAC), on bacterial profiles remains poorly characterized. 

### Aim

The aim of the study was to investigate the correlations between bacterial presence in sinus and PAL samples with demographic, clinical, endoscopic and radiological variables and to assess the influence of OAC on microbial concordance between PAL and MS by focusing on molecular-level analysis using polymerase chain reaction (PCR).

## 2. Materials and Methods

### 2.1. Study Design and Setting

This study was designed as an analytical cross-sectional study conducted at a tertiary academic referral center. The study design and reporting adhere to the Strengthening the Reporting of Observational Studies in Epidemiology (STROBE) statement. The completed checklist is available as Supplementary File S1.

### 2.2. Participants

Between 2019 and 2023 consecutive patients presented with the clinical and radiological symptoms of chronic odontogenic sinusitis (ODS) and periapical lesion (PAL) around maxillary molars or premolars were included in the study. All patients were evaluated by both an otolaryngologist and a dental specialist. Otolaryngological examination consisted of medical interview, nasal endoscopy and CT or CBCT imaging of the sinuses and adjacent maxillary dentition. Radiological signs of sinusitis were mucosal changes within the ostiomeatal complex and/or sinuses that exceeded half of their dimension. Dental evaluation of periapical tissues consisted of percussion, palpation, mobility tests, cold pulp testing, and analysis of CT or CBCT scans. 

### 2.3. Eligibility Criteria

Diagnosis of confirmed ODS required fulfillment of the following criteria: clinical presentation consistent with unilateral maxillary sinusitis, radiological evidence of sinus inflammation adjacent to a dental source on CT, identification of odontogenic pathology such as periapical lesion or failed endodontic treatment and intraoperative confirmation of odontogenic origin during surgical intervention.

### 2.4. Exclusion Criteria

Competing etiologies were systematically excluded. In particular, fungal ball was ruled out based on characteristic CT findings (hyperdense intraluminal material, calcifications, lack of aggressive bony destruction) and absence of fungal debris during intraoperative inspection.

Patients with bilateral sinusitis and bilateral PALs and patients with primary immunodeficiency were excluded from the study. Patients with treatable dental conditions and the ones with just maxillary sinus mucosal thickening were also excluded.

### 2.5. Variables

The following demographic and clinical variables were gathered: age, gender, BMI (Body Mass Index) score, ODS laterality, sinusitis symptoms and nasal endoscopy findings according to the modified Lund–Kennedy endoscopic scoring system [18] at initial otolaryngologist visit. Endoscopic assessment included evaluation of mucosal edema, purulent discharge, and middle meatal obstruction. Endoscopic scoring was not performed intraoperatively to avoid bias related to surgical manipulation. Additional recorded data included sinus opacification extent on computed tomography (CT), dental pathology with the endodontic treatment performed and subjective dental symptoms. Dates of initial rhinologist consultation and dental and ESS treatments were recorded.

Information regarding prior antibiotic therapy was recorded for all patients, including antibiotic class and duration when available. Patients with preoperative antimicrobial exposure of one month prior to the surgical intervention and sample collection were excluded from the study given its potential influence on bacterial detection patterns.

### 2.6. Radiological Assessment

Radiological analysis consisted of multiplanar reconstruction of CT or CBCT scans involving paranasal sinuses and maxillary dentition. The PALs were scored according to the Estrela scale [19]. The incidence of alveolar bone expansion (E) or destruction (D) towards the MS above the PAL was also noted.

The Lund–Mackay score was used for classification of radiographic disease severity. Maxillary sinus, anterior ethmoids, posterior ethmoids and frontal and sphenoid sinuses are each graded between 0 and 2 (0 = no abnormality; 1 = partial opacification; 2 = total opacification). Ostiomeatal complex is graded either 0 or 2 (0 = no abnormality; 2 = total opacification). Maximum score is 12 on each side [20,21]. 

The European Position Paper on Rhinosinusitis and Polyps 2020 (EPOS) considers a total Lund–Mackay score of 2 to be clinically significant when due to complete obstruction of 1 sinus [1].

MS was additionally scored with the Zinreich scale [21]. It is a modification of the Lund–Mackay scale that provides additional nuance for scoring the partial state. Each of the paired paranasal sinuses was scored as 0 (no opacification), 1 (1–25%), 2 (26–50%), 3 (51–75%), 4 (76–99%), or 5 (100%). 

### 2.7. Sample Collection

Samples for the Polymerase Chain Reaction (PCR) analysis were collected at the onset of surgery, which was the endoscopic sinus surgery (ESS) with the extraction of the causative tooth. 

After a standard maxillary antrostomy was created under the visualization of a 30 degrees nasal endoscope, a mucosal biopsy was taken from the floor of the MS. After the extraction of the causative tooth was performed, scrapings from the periapical lesion were collected. Both samples were immediately stored in separate 1 mL vials, which were previously filled with DNAgard Tissue and Cells (Biomatrica, San Diego, CA, USA) under sterile conditions, as described previously [22].

Then, all root sockets were checked for the possible presence of oroantral communication (OAC). This was defined as an intraoperative communication between the oral cavity and maxillary sinus confirmed by probing or visualization. In the case of positive testing, closure using the mucosal flaps was performed. 

### 2.8. Microbiological Analysis

#### 2.8.1. DNA Isolation

Biological samples in 0.5 mL DNAgard Tissue and Cells solution were stored at 4 °C. The detailed description of DNA isolation can be found in Kwiatkowska et al. [22]. Briefly, the samples were sonicated in an ultrasonic washer (Polsonic, Warsaw, Poland), treated with freshly prepared lysozyme, lysostaphin and RNase A, lysed in the presence of proteinase K and vortexed in BashingBead Lysis Tubes (Zymo Research, Irvine, CA, USA). One column DNA purification was performed with the DNA Extraction Genomic Mini kit (Blirt, Gdańsk, Poland). QuantiFluor ONE dsDNA System (Promega, Madison, WI, USA) and Quantus Fluorometer (Promega) were used to verify the DNA concentration. Samples were stored at −80 C° prior to quantitative PCR. 

#### 2.8.2. Quantitative PCR (QPCR)

Primer pairs designed to amplify *16S rRNA* gene fragments of *Eikenella corrodens*, *Fusobacterium nucleatum*, *Peptostreptococcus anaerobius*, *Porphyromonas endodontalis*, *Porphyromonas gingivalis*, *Prevotella intermedia*, *Prevotella nigrescens*, *Pseudomonas aeruginosa*, and *Staphylococcus aureus* were described by Kwiatkowska et al. [22]. Detection of *Streptococcus anginosus* group (*S. anginosus*, *S. constellatus*, *S. intermedius*) was done using primers complementary to the *16S rRNA* gene as reported by Olson et al. [23]. A comprehensive description of QPCR with standard curves prepared from genomic DNA of type strains from the German Collection of Microorganisms and Cell Cultures (DSMZ, https://www.dsmz.de/, accessed on 17 August 2021) was provided previously [22]. DNA samples (10 ng) were analyzed in duplicates using Brilliant III Ultra-Fast SYBR Green QPCR Master Mix (Agilent, Santa Clara, CA, USA). The following reaction conditions were employed on the Agilent Aria Mx instrument: 3 min at 95 °C (hot start), 45 cycles of 5 s at 95 °C and 10 s at 60 °C, and a melt curve with a resolution of 0.5 °C and a soak time of 5 s.

Targeted quantitative PCR analysis provided three complementary outputs: detection rate, defined as presence or absence of specific bacterial targets, quantitative bacterial load expressed as genome copy number; and relative proportion calculated only within the predefined targeted panel. Because the assay was restricted to selected odontogenic pathogens, relative proportions do not represent the full sinonasal microbiome but rather the distribution of organisms within the targeted panel. 

Quantitative analysis of QPCR results, including calculating the number of bacterial genomes was done previously [22]. The results were then converted into percentages of the total due to the unequal loading of microbial DNA in QPCR (human DNA was not removed from the samples). Additionally, the findings were also presented indicating the number of patients with bacteria present in periapical lesions and sinus samples (P1S1), solely in PAL samples (P1S0), only in sinus samples (P0S1), and in neither (P0S0).

In addition to microbiological evaluation, all patients were managed postoperatively according to a standardized multidisciplinary treatment protocol involving otolaryngology and dental/oral–maxillofacial teams [2,24,25]. Empiric antimicrobial therapy was initiated after the surgery with broad-spectrum coverage targeting anaerobic and oral flora and subsequently adjusted based on PCR or culture findings when clinically relevant. The presence of OAC influenced both surgical planning and postoperative antibiotic duration.

#### 2.8.3. Bias

To minimize selection bias, consecutive eligible patients were included. Laboratory personnel were blinded to clinical severity data during qPCR analysis.

#### 2.8.4. Study Size

Given the exploratory nature and rarity of surgically confirmed ODS, all eligible patients within the study period were included.

### 2.9. Statistical Analysis

Statistical analysis was performed with Statistica v. 13 (TIBCO Software Inc., Palo Alto, CA, USA). Data visualizations were performed with Statistica or GraphPad Prism ver. 10.3.0 for Windows (GraphPad Software, Boston, MA, USA, www.graphpad.com, accessed on 5 May 2025). In the case of quantitative variables, basic measures of location and dispersion were given as mean, standard deviation and range. Compliance of the quantitative variables’ distribution with the normal distribution was checked using the Shapiro–Wilk test. In the case of qualitative variables, percentage distributions were given.

The Mann–Whitney U-Test was used to check whether there was a difference in age, BMI, OHIP-14 and SNOT-22 scores between genders. 

Statistical analysis was also performed to evaluate associations between demographic, clinical, and radiological parameters, and the presence of bacterial species in sinus mucosal biopsies. Bacterial detection was treated as a categorical variable (presence/absence), and continuous variables were categorized. Analyses were performed separately for each bacterial species. Associations between categorical variables were assessed using contingency tables. The Pearson chi-square test was used for comparisons involving three groups, while Fisher’s exact test was applied for comparisons between two groups. All tests were two-tailed. To account for multiple hypothesis testing, *p*-values were adjusted using the Bonferroni correction. The family-wise error was controlled at α = 0.05 by dividing α by the number of comparisons. Effect sizes were expressed as odds ratios (OR) with 95% confidence intervals. ORs were derived from 2 × 2 tables, with a Haldane–Anscombe continuity correction applied when zero cell counts were present.

## 3. Results

In total, 28 patients with odontogenic sinusitis (ODS) were included in the study, among which 12 were females (43%) and 16 males (57%). The detailed demographic, clinical, radiological and endoscopic findings of the cohort (total and split by gender) are given in Table 1 and Table 2.

**Figure 1:**
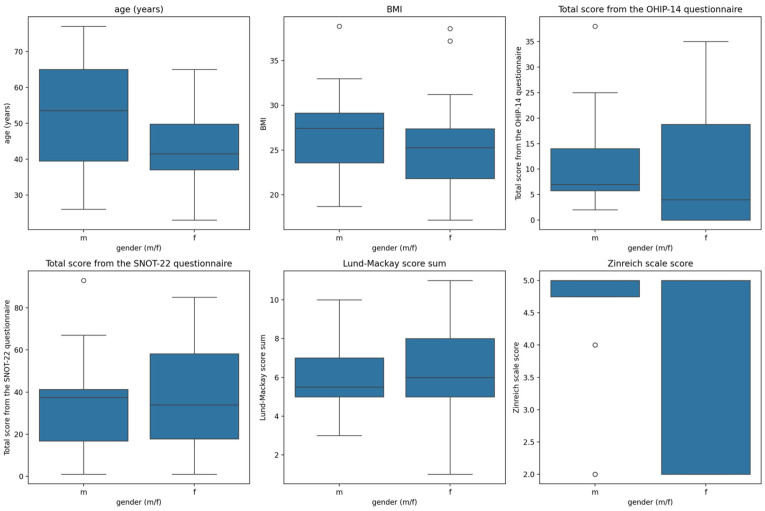
Distribution of age, Body Mass Index (BMI), Total score of OHIP-14—Oral Health Impact Profile-14 and SNOT-22—Sino-Nasal Outcome Test-22 questionnaires, total score on radiological Lund–Mackay scale and score of maxillary sinus opacification presented as total score on Zinreich scale among genders. (m—males, f—females).

Mean patient age was 49.3 (SD = 10.65, Min = 28, Max = 77). Males (52.38 ± 15.37 years) tended to be older than females (42.92 ± 11.43 years), but this difference was not statistically significant (*p* = 0.114). The Men Body Mass Index (BMI) score was 26.6 (SD = 4.35, Min = 17.19, Max = 34.92). Males (27.07 ± 4.87) had slightly higher BMI than females (25.88 ± 6.77), but this was not significantly different (*p* = 0.341). The mean total OHIP-14 score was 11.05 (SD = 7.14, Min = 1.5, Max = 26.5), and the mean total SNOT-22 score was 35.47 (SD = 16.75, Min = 5, Max = 80). OHIP-14 and SNOT-22 questionnaire scores show similar distributions between genders: for OHIP-14: Males (11.00 ± 9.48) vs. Females (10.58 ± 13.66) with *p* = 0.234, and for SNOT-22: Males (34.38 ± 23.89) vs. Females (37.00 ± 25.46), *p* = 0.853. Figure 1 visualizes the distributions of the measured clinical parameters, imaging scores, and quality-of-life questionnaire results and differences between genders. 

There were no statistically significant correlations between age and the other clinical variables.

The bacterial profiles in samples collected from ODS patients were assessed using Polymerase Chain Reaction (PCR) with primers complementary to 16S rRNA gene sequences of selected taxa [22]. In total, 28 samples from MS mucosa (15 on the left side, and 13 on the right side), and 31 samples from PAL were analyzed. The higher number of PAL samples compared to sinus samples was due to the extraction of three causative teeth with PAL from one patient, and two causative teeth from another. The main causative teeth were maxillary molars: in 16 cases (52%) it was first maxillary molar, in 13 cases (42%)—second maxillary molar, and in 2 cases (6%)—second maxillary premolar.

**Figure 2:**
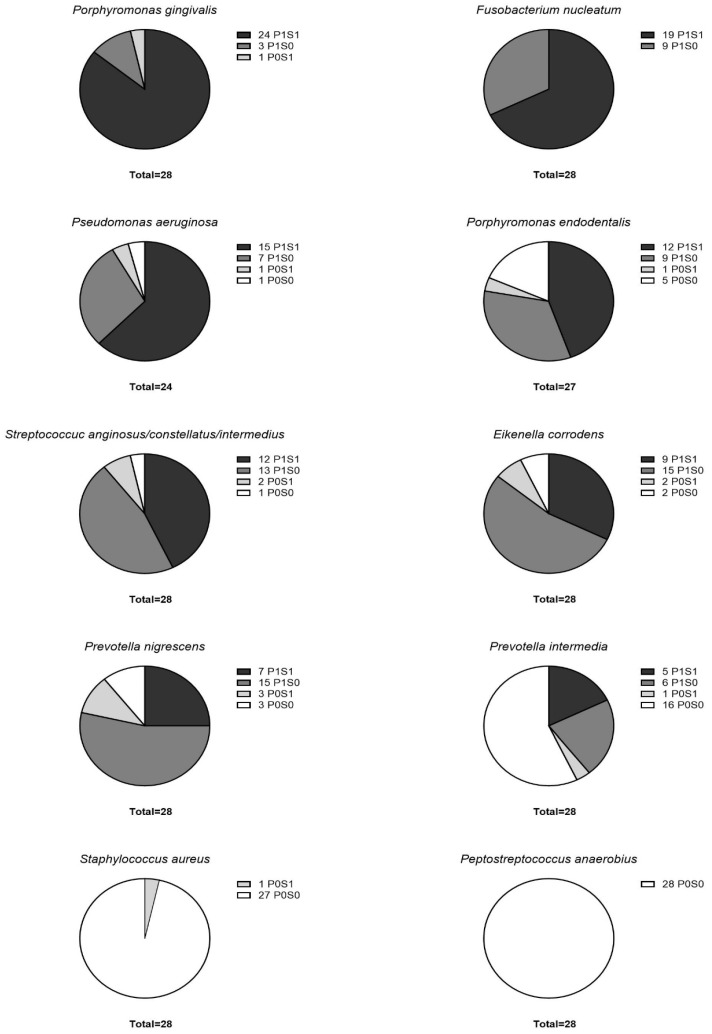
Presence of bacterial taxa in patients’ samples expressed as the number of patients with bacteria present in periapical lesions and sinus samples (P1S1), solely in PAL samples (P1S0), only in sinus samples (P0S1), and in neither (P0S0).

**Figure 3:**
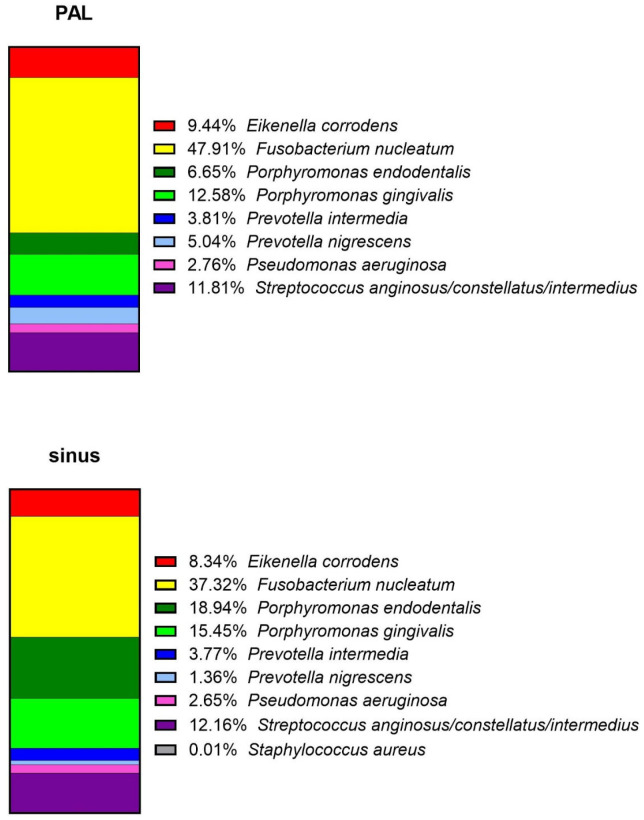
Relative abundance of ten bacterial taxa measured by QPCR in periapical lesions (PAL) and sinus samples.

The highest detection rate of bacteria detected in both the PALs and sinus biopsies of ODS patients was found for *P. gingivalis* and *Fusobacterium* taxa, with 24 double-positive cases out of 28 patients and 19 out of 28, respectively (Figure 2). They were followed by *P. aeruginosa* (15 double-positive cases out of 24 analyzed patients), *P. endodontalis* (12 out of 28 patients) and *S. anginosus* group (12 out of 28 patients). *P. anaerobius* was not detected in any of the analyzed samples, and *S. aureus* was only found in one mucosal biopsy of the sinus. *S. anginosus* group, *E. corrodens*, *P. nigrescens* and *P. intermedia* were more frequently detected exclusively in the PAL samples than in both sinus and PAL samples (Figure 3).

Results from PCR analysis were also analyzed quantitatively. Relative abundances were calculated by dividing the number of genomes of a specific taxon by the cumulative number of bacterial genomes detected in samples. Importantly, these values represent only proportions among the detected taxa analyzed by QPCR and do not reflect absolute bacterial composition. *F. nucleatum* and related *Fusobacterium* species from the human oral cavity and aerodigestive tract were the most abundant among the detected taxa in analyzed PAL (47.91% ± 19.38%) and sinus samples (37.32% ± 32.90%). However, the percentages varied widely among the samples, with a minimal value of 10.83% and a maximal value of 100% for PAL, and 0% and 94.67% for sinus, respectively (Figure 2). In general, the mean percentage results for the analyzed bacteria were comparable between the PAL and the sinus samples. The highest difference could be observed for *P. endodontalis*, with 6.65% ± 11.51% frequency in PAL and 18.94% ± 26.01% in sinus. In contrast, *P. nigrescens* was more frequent in PAL samples (5.04% ± 5.12%) than in sinus samples (1.36% ± 2.87%). 

**Figure 4:**
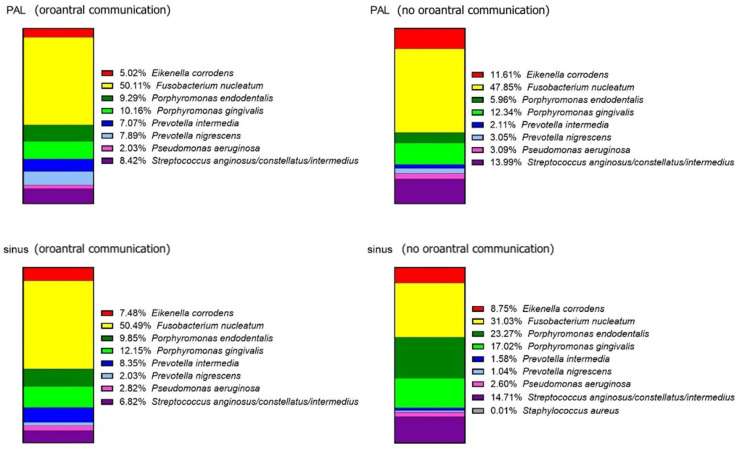
Relative abundance of ten bacterial taxa measured by QPCR in periapical lesions (PAL) and sinus samples in patients with and without oroantral communication (OAC) confirmed during the surgery.

The cohort was also divided into subgroups with or without OAC presence confirmed during the surgery. OAC-positive patients exhibited higher concordance between bacterial profiles in sinus and periapical lesion samples. Variations in the relative proportions of *F. nucleatum*, *P. endodontalis*, and *P. intermedia* were noted between OAC-positive and OAC-negative patients (Figure 4).

The study group was also analyzed for association between demographic, clinical, and radiological parameters and the presence of bacterial species in sinus mucosal biopsies. Due to the multiple comparisons (n = 9), the Boferroni correction was applied and the significance level was set at α = 0.005. *P. anaerobius* was not included in the analysis due to its absence from all the samples analyzed. No significant differences were found between genders for any of the analyzed species (*p* > 0.05, Fisher’s exact test). Age groups (<65 years vs. ≥65 years) similarly showed no significant variations in bacterial detection rates. Body mass index (BMI) categories (<25, 25–30, ≥30) also demonstrated no significant association with bacterial presence. 

The severity of sinus opacification, measured by the Zinreich scale, was assessed for its relationship with bacterial prevalence. A Zinreich score of ≥5, indicating total maxillary sinus opacification, was significantly (*p* = 0.001) associated with *Streptococcus anginosus* group detection, with the odds ratio of 50.09 (95% CI: 2.47–1014.68). Other bacterial species, including *F. nucleatum* and *P. endodontalis*, showed trends towards higher prevalence in patients with more severe radiological findings, though these were not statistically significant. The detailed data with statistical analysis of the abovementioned parameters is given in Table 3.

The type of discharge observed in nasal endoscopy showed significant association with specific bacterial species. Among nine tests performed, three yielded *p*-values below 0.05 (Table 4). However, only the associations for *P. endodontalis* (*p* = 0.005) and the *Streptococcus anginosus* group (*p* = 0.001) remained statistically significant when evaluated against the Bonferroni-corrected significance level. Both bacterial taxa were more frequently detected in patients with thick, purulent discharge, with the odds ratio for *P. endodontalis* equal to 16.67 (95% CI: 2.27–122.22), and 30.00 for *Streptococcus anginosus* group (95% CI: 3.56–252.98). This suggests a potential involvement of these taxa in severe mucosal inflammation.

Other bacteria, including *Fusobacterium nucleatum* and *P. aeruginosa*, showed higher detection frequency in purulent cases, but the differences were not statistically significant. In contrast, patients with clear, thin discharge exhibited lower bacterial detection rates overall, with no specific pathogen predominating. The presence of analyzed bacterial taxa with its association to nasal endoscopy findings is given in Table 4.

PCR-based microbiological analysis demonstrated polymicrobial anaerobe-dominant profiles consistent with odontogenic infection. These findings supported continuation of broad anaerobic coverage and informed targeted antimicrobial adjustments in selected patients. However, given the limited cohort size and wide confidence intervals, these results should be interpreted as exploratory. Patients with confirmed OAC showed higher concordance between sinus and periapical bacterial profiles, reinforcing the concept of direct anatomical spread and guiding simultaneous sinus and dental intervention. Clinical improvement was observed during follow-up; however, outcomes were not evaluated using predefined objective criteria, and therefore these observations should be interpreted cautiously. No patient required revision surgery for persistent infection.

## 4. Discussion

The study presents the results of microbiological and clinical correlation in ODS of endodontic origin. A multidisciplinary approach for the inclusion and exclusion of patients was used throughout the research [2], and to the best of our knowledge, this is the only existing study that compared the PCR results from maxillary sinus and periapical lesion in a cohort of clinically confirmed disease. 

Although it is proven that ODS patients maintain an intact mucosal barrier with sinus mucosa actively fighting underlying dental and sinus infection, the overall bacterial load and dominant bacterial species are factors on which the success of inflammatory response depends [8].

Notably, some findings align with previous research indicating that ODS is most associated with anaerobic flora [4,5,6,11], yet they add nuance regarding the specific bacterial profiles and their correlations with clinical severity.

It was found that a high Zinreich score (≥5), indicative of total maxillary sinus opacification, was significantly associated with the detection of *Streptococcus* species (specifically *anginosus*, *constellatus*, or *intermedius*) (*p* = 0.001). This finding reinforces earlier studies [6] that have reported the prominent role of *Streptococcus* spp. in sinus inflammatory conditions. However, while most literature had focused on their role in acute sinusitis, the presented work extends these observations to chronic ODS associated with endodontic disease, thereby underscoring the pathogenic potential of these organisms in a broader clinical spectrum. 

Studies demonstrate a significant overlap, yet distinct characteristics in the microbial communities of nasal and oral cavities in ODS patients. A systematic review identified *Peptostreptococcus* and *Streptococcus constellatus* as key anaerobic contributors to ODS, with oral samples showing higher microbial diversity compared to sinus samples [11].

The microbiome of ODS predominantly comprises anaerobic bacteria, reflecting its dental origin. Commonly reported bacteria include *Fusobacterium nucleatum*, *Porphyromonas gingivalis*, and *Prevotella* species. These species are associated with periodontal disease and periapical infections [11,12].

As the maxillary premolars and the mesiobuccal roots of maxillary molars exhibit the highest rates of multiple apical foramina, if no treatment is provided for pulp necrosis, the apical tissue might become infected over time [26]. The unique microbial profile of endodontic infection, combined with the innate and adaptive immune response, is thought to influence the progression and severity of ODS [27]. Still, it has not been meticulously studied why only a fraction of patients with maxillary molar endodontic disease develops ODS, if PALs are generally prevalent in the global population [27,28,29].

While PCR-based methods enhance detection of polymicrobial anaerobic flora, their clinical value lies in integration with anatomical and surgical findings. In the presented series, PCR results supported the odontogenic origin of infection and justified prolonged anaerobe-active antimicrobial therapy. Importantly, concordance between sinus and periapical microbiological profiles in patients with OAC suggests direct pathogen transmission across disrupted anatomical barriers. From a clinical standpoint, these findings reinforce the need for simultaneous sinonasal drainage and dental source control rather than isolated antimicrobial treatment. Moreover, microbiological complexity alone did not dictate clinical severity; instead, disease extent, anatomical disruption, and presence of extrasinus spread were more closely associated with outcomes. Thus, PCR should be interpreted as an adjunct to—not a replacement for—clinical and radiological assessment in the management of ODS.

The presented research demonstrated a statistically significant association between purulent discharge on nasal endoscopy and the presence of *Streptococcus anginosus* group (*p* = 0.001) and *Porphyromonas endodontalis* (*p* = 0.005), and the prevalence was high in both sinus and PAL’s biopsies. 

*Prevotella nigrescens* was significantly more frequent in PAL samples (adjusted *p* = 0.019), suggesting that there may be distinct microbial microenvironments at different anatomical sites, as the bacterial communities in PALs may differ subtly from those in the adjacent sinus regions [30].

Given the relatively small sample size and the number of comparisons performed, several associations yielded large odds ratios with wide confidence intervals. These findings should therefore be interpreted cautiously and considered hypothesis-generating rather than confirmatory.

The mechanisms by which predominantly anaerobic odontogenic microbiota establish infection within the relatively oxygen-rich maxillary sinus environment remain incompletely understood. Direct translocation from dental sources through inflamed mucosa or adjacent alveolar bone has been proposed as a principal pathway of spread, initially described by Brook [6,31] and supported by more recent molecular studies demonstrating similar sinus bacterial profiles in odontogenic sinusitis regardless of the presence of OAC [32]. Previous investigations evaluating the role of OAC in facilitating microbial migration between the oral cavity and maxillary sinus have produced heterogeneous results, and most studies rely on radiological or postoperative identification rather than direct intraoperative assessment [9,10,28,33,34,35].

In contrast, the present study evaluated the presence of OAC intraoperatively during ESS and dental extraction combined, allowing precise anatomical confirmation at the time of active infection and specimen collection. This approach, which remains infrequently reported in the literature, enabled simultaneous assessment of sinonasal and odontogenic compartments under controlled surgical conditions. Although no statistically significant differences in overall microbial profiles were observed between patients with and without OAC, cases with confirmed intraoperative communication demonstrated a higher degree of concordance between bacterial findings in MS mucosa and PAL samples. This trend supports the concept that direct anatomical communication may facilitate microbial continuity, even if not strictly required for odontogenic spread.

The use of PCR to analyze bacterial presence and loads in ODS may offer some advantages over traditional microbiological methods. PCR enables the detection of specific bacterial DNA with high sensitivity and specificity, which is particularly beneficial for identifying fastidious and anaerobic organisms that are difficult to culture [9,10]. While molecular diagnostics improved pathogen detection, this study was not designed to evaluate treatment modification or clinical outcomes based on PCR findings.

There are some limitations to PCR-based methods. While it offers high sensitivity, it does not always provide information on the viability or metabolic activity of the detected organisms. Furthermore, the complexity of the microbiome in ODS, with its polymicrobial nature, means that PCR-based methods may detect multiple bacteria, but the clinical significance of each organism and its potential interactions are not fully understood. Also, PCR’s reliance on sequence-specific primers can miss non-target species or detect species in very low quantities that may not play a significant role in the disease. This highlights the need for integrative approaches, combining PCR with clinical examination and advanced imaging techniques such as CBCT to more accurately diagnose and manage ODS. 

## 5. Limitations and Future Directions

While this study identifies significant bacterial associations, the absence of detectable *Peptostreptococcus anaerobius* and the low prevalence of *Staphylococcus aureus* suggest variability in the microbial profile of ODS. This variability may reflect individual differences in oral health, immune responses, or treatment histories. The relatively small cohort size and multiple statistical comparisons increase the risk of overestimating effect sizes. Several associations were characterized by wide confidence intervals, indicating limited precision. Therefore, the findings should be regarded as exploratory and require validation in larger, multicenter cohorts.

Also, targeted QPCR restricts analysis to predefined organisms and does not provide full microbiome profiling. Future research should employ larger cohorts and integrate high-throughput sequencing to uncover broader microbial diversity and functional insights into ODS pathogenesis. Additionally, the lack of statistically significant differences in bacterial prevalence across demographic groups such as age and BMI highlights the need for more analyses to explore potential influences of systemic health on ODS microbiology.

Given that certain bacterial species have a strong association with severe clinical presentations, there is potential for developing more targeted antimicrobial therapies. By identifying patients at risk due to the dominance of pathogenic bacteria, clinicians might implement proactive measures (for example: early surgical intervention, tailored pharmacotherapy) to prevent further development of the disease. 

## 6. Conclusions

This study underscores the polymicrobial nature of ODS, dominated by anaerobes, with specific bacteria correlating to clinical severity markers like discharge type and radiological findings. The observed concordance in bacterial profiles between sinus and PALs—particularly in cases with OAC—supports the hypothesis that odontogenic infections can spread more directly into the MS. This reinforces the role of dental etiology in the development of sinusitis and underscores the importance of a multidisciplinary approach in managing these infections, involving both dental and ENT specialists.

QPCR demonstrated high sensitivity in detecting odontogenic pathogens and may represent a promising adjunctive diagnostic tool. By identifying patients at risk due to the dominance of pathogenic bacteria, clinicians might implement proactive measures (e.g., early surgical intervention, tailored pharmacotherapy) to prevent complications such as more advanced chronic sinusitis or persistent OAC. Integration of PCR-based microbiological analysis with clinical presentation, imaging findings, and surgical management may complement therapeutic decision-making but should always be interpreted within a multidisciplinary clinical framework.

## Figures and Tables

**Table 1:** Demographics and clinical parameters of the analyzed cohort (BMI—Body Mass Index, OHIP-14—Oral Health Impact Profile-14, SNOT-22—Sino-Nasal Outcome Test-22).

	Age (Years) Mean (Min–Max)	BMI Mean (Min–Max)	OHIP-14 Total Score Mean (Min–Max)	SNOT-22 Total Score Mean (Min–Max)	Polyps on Nasal Endoscopy	Purulent Discharge on Nasal Endoscopy	Edema on Nasal Endoscopy	Previous Endodontic Treatment	Confirmed Oroantral Communication During Surgery
Total	48.32 (23–77)	26.56 (17.19–38.82)	10.82 (0–38)	35.5 (1–93)	5/28 (18%)	14/28 (50%)	26/28 (93%)	18/28 (64%)	8/28 (29%)
Males	52.38 (26–77)	27.07 (17.19–38.82)	11 (2–38)	34.38 (1–93)	3/16 (19%)	9/16 (56%)	15/16 (94%)	9/16 (56%)	4/16 (25%)
Females	42.92 (23–65)	25.88 (17.19–38.57)	10.58 (0–35)	37 (1–85)	2/12 (17%)	5/12 (42%)	11/12 (92%)	9/12 (75%)	4/12 (33%)

**Table 2:** Radiological parameters of the analyzed cohort stated in the computed tomography (CT) or cone-beam computed tomography images (CBCT). None of the parameters showed statistically significant differences between genders (all *p*-values > 0.05).

	Maxillary Sinus Ostium Obstruction on CT	Zinreich Scale Score	Lund-Mackay Score Sum	Maxillary Sinus Total Opacification	Frontal Ethmoids Total Opacification	Posterior Ethmoids Total Opacification	Frontal Sinus Total Opacification	Sphenoid Sinus Total Opacification	Estrela Scale Sum	Alveolar Bone Destruction on CT
Total	24/28 (86%)	4.21 (2–5)	6.21 (1–11)	21/28 (75%)	10/28 (36%)	4/28 (14%)	5/28 (18%)	0/28 (0%)	3.75 (2–5)	21/28 (75%)
Males	14/16 (88%)	4.5 (2–5)	6.13 (3–10)	13/16 (81%)	4/16 (25%)	1/16 (6%)	2/16 (13%)	0/16 (0%)	3.81 (2–5)	13/16 (81%)
Females	10/12 (83%)	3.83 (2–5)	6.33 (1–11)	8/12 (67%)	6/12 (50%)	3/12 (25%)	3/12 (25%)	0/12 (0%)	3.67 (2–5)	8/12 (67%)

**Table 3:** Analysis of the association between bacteria detected in sinus mucosal biopsies by PCR and various clinical and radiological variables. BMI—Body Mass Index, NA—not applicable. Statistical significance was defined as α = 0.005 (Bonferroni-adjusted); bold values are statistically significant.

Bacteria Detected with the Use of PCR Method	Female	Male	Fisher’s Exact Test (*p* Value)	Age < 65 Years	Age ≥ 65 Years	Fisher’s Exact Test (*p* Value)	BMI < 25	BMI ≥ 25 <30	BMI ≥ 30	Chi Square Test (*p* Value)	Zinreich Score < 5	Zinreich Score ≥ 5	Fisher’s Exact Test (*p* Value)
*Eikenella corrodens* (0—absent)	7	10		12	5		7	7	3		6	11	
*Eikenella corrodens* (1—present)	5	6	1.000	10	1	0.355	3	5	3	0.712	3	8	1.000
*Fusobacterium nucleatum* (0—absent)	3	6		8	1		3	3	3		5	4	
*Fusobacterium nucleatum* (1—present)	9	10	0.687	14	5	0.630	7	9	3	0.555	4	15	0.097
*Peptostreptococcus anaerobius* (0—absent)	12	16		22	6		10	12	6		9	19	
*Peptostreptococcus anaerobius* (1—present)	0	0	NA	0	0	NA	0	0	0	NA	0	0	NA
*Porphyromonas endodentalis* (0—absent)	7	7		11	3		5	7	2		7	7	
*Porphyromonas endodentalis* (1—present)	5	8	0.704	10	3	1.000	5	4	4	0.484	2	11	0.103
*Porphyromonas gingivalis* (0—absent)	2	1		3	0		2	1	0		2	1	
*Porphyromonas gingivalis* (1—present)	10	15	0.560	19	6	1.000	8	11	6	0.429	7	18	0.234
*Prevotella intermedia* (0—absent)	10	12		17	5		9	9	4		8	14	
*Prevotella intermedia* (1—present)	2	4	0.673	5	1	1.000	1	3	2	0.504	1	5	0.630
*Prevotella nigrescens* (0—absent)	7	11		14	4		6	8	4		7	11	
*Prevotella nigrescens* (1—present)	5	5	0.698	8	2	1.000	4	4	2	0.940	2	8	0.417
*Pseudomonas aeruginosa* (0—absent)	4	4		6	2		5	2	1		3	5	
*Pseudomonas aeruginosa* (1—present)	6	10	0.673	13	3	1.000	3	8	5	0.100	4	12	0.647
*Staphylococcus aureus* (0—absent)	11	16		21	6		9	12	6		9	18	
*Staphylococcus aureus* (1—present)	1	0	0.429	1	0	1.000	1	0	0	0.393	0	1	1.000
*Streptococcus anginosus*/*constellatus*/*intermedius* (0—absent)	6	8		11	3		7	5	2		9	5	
*Streptococcus anginosus*/*constellatus*/*intermedius* (1—present)	6	8	1.000	11	3	1.000	3	7	4	0.273	0	14	**0.001**

**Table 4:** Presence or absence of bacteria detected with the use of PCR and its association with the nasal endoscopy findings with the *p*-values from Fisher’s exact test. Statistical significance was defined as α = 0.005 (Bonferroni-adjusted). Odds ratio (OR) with 95% confidence interval (CI) was used to identify bacterial taxa associated with thick, purulent discharge. NA—not applicable.

Bacteria Detected with the Use of PCR Method	Clear, Thin Discharge	Thick, Purulent Discharge	Fisher’s Exact Test (*p* Value)	OR (95% CI)
*Eikenella corrodens* (0—absent)	8	7		
*Eikenella corrodens* (1—present)	4	7	0.453	2.00 (0.41–9.84)
*Fusobacterium nucleatum* (0—absent)	6	1		
*Fusobacterium nucleatum* (1—present)	6	13	0.026	13.00 (1.27–133.29)
*Peptostreptococcus anaerobius* (0—absent)	12	14		
*Peptostreptococcus anaerobius* (1—present)	0	0	NA	0.86 (0.02–46.71)
*Porphyromonas endodentalis* (0—absent)	10	3		
*Porphyromonas endodentalis* (1—present)	2	10	0.005	16.67 (2.27–122.22)
*Porphyromonas gingivalis* (0—absent)	2	0		
*Porphyromonas gingivalis* (1—present)	10	14	0.203	6.90 (0.30–159.30)
*Prevotella intermedia* (0—absent)	11	9		
*Prevotella intermedia* (1—present)	1	5	0.170	6.11 (0.60–62.23)
*Prevotella nigrescens* (0—absent)	9	7		
*Prevotella nigrescens* (1—present)	3	7	0.248	3.00 (0.56–16.01)
*Pseudomonas aeruginosa* (0—absent)	5	1		
*Pseudomonas aeruginosa* (1—present)	5	11	0.056	11.00 (1.00–120.43)
*Staphylococcus aureus* (0—absent)	11	14		
*Staphylococcus aureus* (1—present)	1	0	0.462	0.26 (0.01–7.12)
*Streptococcus anginosus*/*constellatus*/*intermedius* (0—absent)	10	2		
*Streptococcus anginosus*/*constellatus*/*intermedius* (1—present)	2	12	0.001	30.00 (3.56–252.98)

## Data Availability

The original contributions presented in this study are included in the article/Supplementary Material. Further inquiries can be directed to the corresponding author.

## Supplementary Materials

The following supporting information can be downloaded at: https://www.mdpi.com/article/10.3390/jcm15051814/s1, File S1: STROBE Statement—Checklist of Items that Should Be Included in Reports of Cross-Sectional Studies.

## Abbreviations

The following abbreviations are used in this manuscript:

ODS	Odontogenic sinusitis
CRS	Chronic Rhinosinusitis
CT	Computed tomography
CBCT	Cone-beam computed tomography
PAL	Periapical lesion
RCT	Root-canal treatment
ESS	Endoscopic sinus surgery
MS	Maxillary Sinus
PCR	Polymerase Chain Reaction
OAC	Oroantral communication

## References

[1] Fokkens W.J., Lund V.J., Hopkins C., Hellings P.W., Kern R., Reitsma S., Toppila-Salmi S., Bernal-Sprekelsen M., Mullol J., Alobid I. (2020). European Position Paper on Rhinosinusitis and Nasal Polyps 2020. Rhinology.

[2] Craig J.R. (2022). Odontogenic sinusitis: A state-of-the-art review. World J. Otorhinolaryngol.—Head Neck Surg..

[3] Goyal V.K., Ahmad A., Turfe Z., Peterson E.I., Craig J.R. (2021). Predicting Odontogenic Sinusitis in Unilateral Sinus Disease: A Prospective, Multivariate Analysis. Am. J. Rhinol. Allergy.

[4] Workman A.D., Granquist E.J., Adappa N.D. (2018). Odontogenic sinusitis: Developments in diagnosis, microbiology, and treatment. Curr. Opin. Otolaryngol. Head Neck Surg..

[5] Saibene A.M., Vassena C., Pipolo C., Trimboli M., De Vecchi E., Felisati G., Drago L. (2016). Odontogenic and rhinogenic chronic sinusitis: A modern microbiological comparison. Int. Forum Allergy Rhinol..

[6] Brook I. (2005). Microbiology of acute and chronic maxillary sinusitis associated with an odontogenic origin. Laryngoscope.

[7] Legert K.G., Zimmerman M., Stierna P. (2004). Sinusitis of odontogenic origin: Pathophysiological implications of early treatment. Acta Oto-Laryngol..

[8] Craig J.R., Hopkins C. (2024). Sinus Pathophysiology of Odontogenic Sinusitis. Otolaryngol. Clin. N. Am..

[9] Haider A.A., Marino M.J., Yao W.C., Citardi M.J., Luong A.U. (2019). The Potential of High-Throughput DNA Sequencing of the Paranasal Sinus Microbiome in Diagnosing Odontogenic Sinusitis. Otolaryngol.—Head Neck Surg..

[10] Mussano F., Ferrocino I., Gavrilova N., Genova T., Dell’Acqua A., Cocolin L., Carossa S. (2018). Apical periodontitis: Preliminary assessment of microbiota by 16S rRNA high throughput amplicon target sequencing. BMC Oral Health.

[11] Areizaga-Madina M., Pardal-Peláez B., Montero J. (2023). Microbiology of Maxillary Sinus Infections: Systematic Review on the Relationship of Infectious Sinus Pathology with Oral Pathology. Oral.

[12] Wu J., Zheng M., Zhao Y., Yin W., Sima Y., Zhao J., Wang X., Lin J., Zhang L. (2023). Bacterial diversity and community characteristics of the sinus and dental regions in adults with odontogenic sinusitis. BMC Microbiol..

[13] Puglisi S., Privitera S., Maiolino L., Serra A., Garotta M., Blandino G., Speciale A. (2011). Bacteriological findings and antimicrobial resistance in odontogenic and non-odontogenic chronic maxillary sinusitis. J. Med. Microbiol..

[14] Yusufoglu S.I., Erbasar G.N.H., Gülen O. (2021). Evaluation of the effect of periapical lesions and other odontogenic conditions on maxillary sinus mucosal thickness characteristics and mucosal appearance: A cbct study. J. Dent. Res. Dent. Clin. Dent. Prospect..

[15] Yeung A.W.K., Hung K.F., Li D.T.S., Leung Y.Y. (2022). The Use of CBCT in Evaluating the Health and Pathology of the Maxillary Sinus. Diagnostics.

[16] Nair U.P., Nair M.K. (2010). Maxillary sinusitis of odontogenic origin: Cone-beam volumetric computerized tomography-aided diagnosis. Oral Surg. Oral Med. Oral Pathol. Oral Radiol. Endodontol..

[17] Kwiatkowska M., Szczygielski K., Skrzypiec Ł., Jurkiewicz D. (2024). Predictive value of tooth and sinuses radiological characteristics in managing odontogenic sinusitis of endodontic origin. Otolaryngol. Pol..

[18] Psaltis A.J., Li G., Vaezeafshar R., Cho K.S., Hwang P.H. (2014). Modification of the Lund-Kennedy endoscopic scoring system improves its reliability and correlation with patient-reported outcome measures. Laryngoscope.

[19] Panzarella F.K., Coelho M.S., Estrela C. (2023). Association between odontogenic conditions and maxillary sinus abnormalities. Ann. Palliat. Med..

[20] Likness M.M., Pallanch J.F., Sherris D.A., Kita H., Mashtare T.L., Ponikau J.U. (2014). Computed tomography scans as an objective measure of disease severity in chronic rhinosinusitis. Otolaryngol.—Head Neck Surg..

[21] Snidvongs K., Dalgorf D., Kalish L., Sacks R., Pratt E., Harvey R.J. (2014). Modified Lund Mackay Postoperative Endoscopy Score for defining inflammatory burden in chronic rhinosinusitis. Rhinology.

[22] Kwiatkowska M.A., Trębińska-Stryjewska A., Andrejuk K., Jurkiewicz D., Trafny E.A., Guzek A. (2025). Comparative Approaches to Microbial Profiling in Odontogenic Sinusitis with Periapical Lesions. https://www.researchsquare.com/article/rs-8067667/v1.

[23] Ordinola-Zapata R., Azevedo B., Tataryn R.W., Versiani M.A. (2024). Maxillary Dental Anatomy and Physiology: Endodontic and Periodontal. Otolaryngol. Clin. N. Am..

[24] Silva E.J.N.L., Pinto K.P., Versiani M.A., Sassone L.M. (2024). Dental Pathophysiology of Odontogenic Sinusitis: Endodontic Infections. Otolaryngol. Clin. N. Am..

[25] Nunes C.A.B.C.M., Guedes O.A., Alencar A.H.G., Peters O.A., Estrela C.R.A., Estrela C. (2016). Evaluation of Periapical Lesions and Their Association with Maxillary Sinus Abnormalities on Cone-beam Computed Tomographic Images. J. Endod..

[26] Dumitrescu A., Martu M.A., Nemtoi A., Sirghe A., Chelaru L., Tatarciuc D., Dumitrescu A.-M., Haba D. (2021). Association between cone-beam computed tomography and histological and immunohistochemical features in periapical lesions correlated with thickened maxillary sinus mucosa. Medicina.

[27] Niazi S.A., Bakhsh A. (2022). Association between Endodontic Infection, Its Treatment and Systemic Health: A Narrative Review. Medicina.

[28] Brook I. (2006). Sinusitis of odontogenic origin. Otolaryngol.—Head Neck Surg..

[29] Yassin-Kassab A., Bhargava P., Tibbetts R.J., Griggs Z.H., Peterson E.I., Craig J.R. (2021). Comparison of bacterial maxillary sinus cultures between odontogenic sinusitis and chronic rhinosinusitis. Int. Forum Allergy Rhinol..

[30] Bravo Cordero G., Minzer Ferrer S., Fernández L. (2016). Odontogenic Sinusitis, Oro-antral Fistula and Surgical Repair by Bichat’s Fat Pad: Literature Review. Acta Otorrinolaringol..

[31] Adamska P., Pylińska-Dąbrowska D., Stasiak M., Kaczoruk-Wieremczuk M., Kozłowska E., Zedler A., Studniarek M. (2024). Treatment of Odontogenic Maxillary Sinusitis with the Use of Growth Factors in Advanced Platelet-Rich Fibrin for Immediate Closure of Oro-Antral Communication: A Case Report. Int. J. Mol. Sci..

[32] Andric M., Saranovic V., Drazic R., Brkovic B., Todorovic L. (2010). Functional endoscopic sinus surgery as an adjunctive treatment for closure of oroantral fistulae: A retrospective analysis. Oral Surg. Oral Med. Oral Pathol. Oral Radiol. Endodontol..

[33] Galli M., De Soccio G., Cialente F., Candelori F., Federici F.R., Ralli M., De Vincentiis M., Minni A. (2020). Chronic maxillary sinusitis of dental origin and oroantral fistula: The results of combined surgical approach in an Italian university hospital. Bosn. J. Basic. Med. Sci..

[34] Sabatino L., Lopez M.A., Di Giovanni S., Pierri M., Iafrati F., De Benedetto L., Moffa A., Casale M. (2023). Odontogenic Sinusitis with Oroantral Communication and Fistula Management: Role of Regenerative Surgery. Medicina.

[35] Craig J.R., Tataryn R.W., Saibene A.M. (2024). The Future of Odontogenic Sinusitis. Otolaryngol. Clin. N. Am..

