# Supplementary material for: Microbiological PCR Characteristics of Odontogenic Sinusitis and Their Clinical Correlates: A Cross-Sectional Analysis
Source: J Clin Med. 2026 Feb 27;15(5):1814. doi: 10.3390/jcm15051814 (PMC12985445; doi:10.3390/jcm15051814)
Supplement: Supplementary file 1 [file jcm-15-01814-s001.zip › jcm-4133961-supplementary.pdf]

Supplementary Material

STROBE Statement—Checklist of Items that Should Be Included in Reports of Cross-Sectional Studies

Section/Topic	Item No.	Recommendation	Reported on Page No.
**Title and Abstract**	1(a)	Indicate the study's design with a commonly used term in the title or abstract	Title page; Abstract (Methods)
	1(b)	Provide in the abstract an informative and balanced summary of what was done and what was found	Abstract
**Introduction**	2	Explain the scientific background and rationale for the investigation	Introduction
	3	State specific objectives, including any prespecified hypotheses	Introduction (final paragraph)
**Methods**	4	Present key elements of study design early in the paper	Methods – Study Design
	5	Describe the setting, locations, and relevant dates	Methods – Study Design and Setting
	6(a)	Give the eligibility criteria and sources/methods of selection of participants	Methods – Participants; Eligibility Criteria
	7	Clearly define all outcomes, exposures, predictors, potential confounders, and effect modifiers	Methods – Variables
	8	For each variable of interest, give sources of data and details of methods of assessment	Methods – Data Sources and Measurement
	9	Describe any efforts to address potential sources of bias	Methods – Bias
	10	Explain how the study size was arrived at	Methods – Study Size
	11	Explain how quantitative variables were handled in the analyses	Methods – Statistical Analysis
	12(a)	Describe all statistical methods, including those used to control for confounding	Methods – Statistical Analysis
	12(b)	Describe any methods used to examine subgroups and interactions	Methods – Statistical Analysis
	12(c)	Explain how missing data were addressed	Methods – Statistical Analysis
	12(d)	If applicable, describe analytical methods taking account of sampling strategy	Not applicable
	12(e)	Describe any sensitivity analyses	Methods – Statistical Analysis
**Results**	13(a)	Report numbers of individuals at each stage of study	Results – Patient Characteristics

Section/Topic	Item No.	Recommendation	Reported on Page No.
	13(b)	Give reasons for non-participation at each stage	Results
	13(c)	Consider use of a flow diagram	Figure 1
	14(a)	Give characteristics of study participants	Results – Baseline Characteristics
	14(b)	Indicate number of participants with missing data for each variable	Results
	15	Report numbers of outcome events or summary measures	Results
	16(a)	Give unadjusted estimates and, if applicable, adjusted estimates with precision (e.g., 95% CI)	Results – Primary and Exploratory Analyses
	16(b)	Report category boundaries when continuous variables were categorized	Methods / Results
	16(c)	If relevant, consider translating estimates of relative risk into absolute risk	Not applicable
	17	Report other analyses done (e.g., subgroup analyses, interactions, sensitivity analyses)	Results – Exploratory Analyses
**Discussion**	18	Summarize key results with reference to study objectives	Discussion – First Paragraph
	19	Discuss limitations of the study	Discussion – Limitations
	20	Provide cautious overall interpretation of results	Discussion
	21	Discuss generalizability (external validity) of the study results	Discussion – Clinical Implications
**Other Information**	22	Give the source of funding and role of funders	Funding Section
